# Improved exosome isolation methods from non-small lung cancer cells (NC1975) and their characterization using morphological and surface protein biomarker methods

**DOI:** 10.1007/s00432-023-04682-6

**Published:** 2023-03-25

**Authors:** Elham O. Mahgoub, Galal M. Abdella

**Affiliations:** 1 Science and Engineering Department, Hamad Bin Khalifa University, P. O. Box 34110, Doha, Qatar; 2 Mechanical and Industrial Engineering Department, Qatar University, P. O. Box 2713, Doha, Qatar

**Keywords:** Exosome, Extracellular vesicle (EVs), Biomarker, Non-small lung cancer cell, NCI1975

## Abstract

This study has demonstrated improved methods for isolating exosomes from non-small lung cancer cells, which address the problems characterized by exosome morphological and chemical methods. To improve the isolation methods, cells from the NCI 1975 cell line were used as the source for exosomes. The isolation processes were carried out using serial isolation techniques in addition to specific preservation tools. The isolated exosomes were characterized using transmission electron microscopy (TEM), and scanning electron microscopy (SEM) was added for further assurance of the investigation results. The statistical analysis results showed that the size distributions of apoptotic vesicles (APV) 450 nm and necrotic bodies (NCB) 280 nm (extracellular vesicles) were significantly different from exosomes (*P* < 0.001). In contrast, the exosome size distribution was not significantly different from the published exosome sizes, as demonstrated by statistical analysis tools. This study confirmed the improved methods for isolating exosomes that make exosomes accessible for use in the diagnosis and prognosis of non-small cell lung cancer (NSCLC).

## Introduction

Exosomes are nanovesicles that are constantly secreted by cells into the human body circulation. Their described sizes vary considerably, which mirrors the difference in the isolation techniques. In this study, we have identified improved methods for isolating exosomes from non-small lung cancer cells. The exosome isolation methods commonly used are differential ultracentrifugation, size exclusion chromatography, and immune affinity. Differential ultracentrifugation is a widely used method for isolating exosomes because of its ease of handling large volumes of conditioned media, thereby improving the yield. However, there are disadvantages to ultracentrifugation-based exosome isolation methods: loss of membrane integrity, low yield, and low purity (Lobb et al. 2015; Van Deun et al. 2014; Momen-Heravi et al. 2013), and (Li et al. 2017). Therefore, adding more methods to maintain purity and keep the yield stable for longer is critical for isolated exosomes. Differential ultracentrifugation followed by ultrafiltration and cryopreservation adds exosome stability.

Generally, isolated exosomes are characterized by their morphology, size, a surface marker protein expression, purity, and yield concentration (Gupta et al. 2018). Western blotting is the standard method for chemically characterizing exosomes collected from cell culture. In addition, due to their familiar cell sources, all exosomes have shared profiling that allows their identification based on different surface protein biomarkers, such as ALIX, TSG101, and CD63. CD81, CD9, EpCAM, or Rab5 (Van Niel et al. 2011). Additionally, precipitation techniques such as the Exosome Isolation Kit from Cell Culture (Invitrogen) are considered an important chemical characterization technique of exosomes (Barrès et al. 2010). Furthermore, a previous study demonstrated that morphological methods such as electron microscopy results of exosome size distribution and concentration depend upon the isolation method used (Gupta et al. 2018; La Shu et al. 2020). As mentioned in many references, differential ultracentrifugation and ultrafiltration yielded up to 58-fold more exosomes than ultracentrifugation alone (Gupta et al. 2018; La Shu et al. 2020). Furthermore, non-small cell lung cancer cells (NSCLC)-secreted exosomes in culture media with the help of endosomes. Cytoplasm preservation methods are used to preserve exosomes in higher purity and stability for more extended periods in a technique called cryopreservation. This method can add protein stability and purity to exosomes, as mentioned in many refs (La Shu et al. 2020, 2021).

The morphological characterization method is usually used to differentiate the shapes and sizes of exosomes in comparison with the size and purity of other types of extracellular vesicles, especially apoptotic vesicles and necrotic bodies. Using two types of electron microscopes to measure the sizes of exosomes and some types of extracellular vehicles is an essential tool when utilizing different statistical approaches to prove the exosome's stability in size and shape after excessive isolation methods. In this study, exosome isolation techniques were improved to increase the purity and maintain constant exosome sizes. Furthermore, that approach was investigated using different morphological methods and proven by statistical analysis results.

## Materials and methods

### Materials

The NCI 1975 cell line was received from the Institute of Translation Medicine (Hamad Corporations), Macrosep® Advance Centrifugal Device (pall, Life Sciences), Nanosep 100 k Omega (pall, Life Sciences), and Pierce BSA Protein Assay Kit (Thermo Fisher Scientific). CD81 antibody, Santa Cruz, goat anti-mouse polyclonal-HRP, Thermo Fisher Scientific), SuperSignal Chemiluminescent Substrates, Thermo Fisher Scientific), Pierce BSA Protein Assay Kit, Thermo Fisher Scientific). Beckman Coulter Avanti J-2615 CP super centrifuge (), Beckman Coulter Optima™ L-80XP ultracentrifuge (Type P55S12-065 rotor (8 ml), (k-factor: 157.7) and Type P55S12-065 rotor (1.5 ml)).

### Methods

#### Cell culture

NCI1975 cells were grown to 70% confluence, and then the cells were washed twice with PBS and 14 ml of DMEM with no serum. NCI1975 cells were incubated for 24 h, and after 48 h, 14 ml of conditioned medium (Meenakshi 2013) was harvested from 70% confluent NCI1975 cells. The conditioned medium was centrifuged at 4000 × g for 10 min to remove detached cells. An amount of 8 ml in each tube of conditional media was centrifuged in a Beckman Coulter Avanti J-2615 CP centrifuge at 15,000 × g at 4 °C for 1 h to remove apoptotic bodies, microvesicles, and cell debris contamination. In addition, a transparent pellet of the exosome was collected.

#### Exosome isolation techniques

##### Differential ultracentrifugation

Additionally, 4 ml of collected supernatant was centrifuged in a Beckman Coulter Optima™L-80XP Ultracentrifuge at 53,000 g rpm at 4 °C for 2 h with a Type P55S12-065 rotor (k-factor: 157.7). The supernatant was carefully removed, and crude exosome-containing pellets were resuspended in 1 mL of ice-cold PBS and pooled. The second round of ultracentrifugation at 53,000 rpm at 4°C for 90 min with a Type P55S12-065 rotor (1.5 ml) was carried out, and the resulting exosome pellet was resuspended in 50 µL of PBS or loading buffer before use in microscopes. Additionally, 50 µL of RIPA buffer was added to samples prepared for the Western blot test. Many rounds of affinity ultracentrifugation was performed to collect enough exosomes resuspended in 5 ml of PBS to be used latter in ultra-filtration.

##### Ultrafiltration devices

The collected exosomes stored in PBS buffer after many rounds of ultracentrifugation were used as starting material of 4 ml. The collected exosomes dissolved in PBS buffer were used to isolate fractions 1 and 2. The process was carried out as follows: a 300 kDa filter was used to collect exosomes from 500 µL of supernatant from a super centrifuge at 12,000 rpm for 10 min. This process was repeated until most of the supernatant went through the filter. The collected fraction on the top is F1, while the flow-through is F2. F1 was stored at  – 80 °C for further analysis. The second fraction of supernatant was filtered through a 100 kDa filter at 12.000 rpm for 10 min. Then, the process was repeated until most of the supernatant went entirely through the filter. The collected fraction on the top is F3, while the flow-through is F4. F3 was stored at  – 80 °C for further analysis. A BCA protein assay kit was used to determine the protein concentration. Additionally, 50 µL of RIPA buffer was added to samples prepared for the Western blot test to observe the solubility and function of exosome proteins.

##### Exosome cryopreservation

Exosomes are stored at  – 80 °C or cryopreserved in liquid nitrogen ( – 196 °C), as mammalian cells are usually held. First, the isolated exosomes were mixed with an equal medium volume containing two DMSO (10%). The mixture was then aliquoted at 1 ml into each cryopreservation tube. Then, the mixture was covered and stored in a  – 80 °C freezer overnight. Finally, the prepared samples were placed in liquid nitrogen for preservation. Exosomes were thawed on ice for 10 min according to recovering cell lines, washed once in 20 ml of PBS, and then resuspended and ultracentrifuged to collect the pellet. Finally, the supernatant was discarded, and the exosome pellet was resuspended in 200 μl of PBS for TEM and SEM analyses. Besides, 50 µL of RIPA buffer was added to samples prepared for the Western blot test.

#### Characterization of exosomes

##### Chemical characterization

Characterization of extracted exosomes using western blot analysis: Protein fraction size was detected using 10% SDS‒PAGE. When the collected exosome was 25 µl (4 mg/ml), lysate protein samples were loaded into each well and run in parallel with Page Ruler Prestained Protein Ladder (Thermo Fisher Scientific) at 100 V for one and a half hours. Next, the gels were blotted onto membranes at 100 V for 1 h. The membranes were then blocked with 10 ml of 1 × blocking buffer (1 ml of 10 × Pierce clear milk plus 9 ml 1 × Fast Wash Buffer, Thermo Fisher Scientific) for 1 h. Then, 10 ml of goat anti-mouse polyclonal-HRP (1:1000, Thermo Fisher Scientific) with 1:1000 CD9 rabbit anti-human primary antibody was added. Then, 1:20.000 Goat anti-rabbit IgG secondary antibody in blocking buffer was added to each membrane and incubated for an hour; the membrane was washed three times with washing buffer. Afterward, the band was developed using Super Signal Chemiluminescent Substrates, Thermo Fisher Scientific) and incubated in the dark for 5 min. Images were taken using an X-ray film CCD imager.

### Morphological characterization

#### Transmission electron microscopy (TEM)

Transmission electron microscopy (JEM-1200-EX-microscope-JEOL) is a morphology characterization method used to characterize exosomes (Akishima, Japan). First, the samples were dissolved in HEPES buffer (4-[2-hydroxyethyl]-1-piperazine ethane sulfonic acid). Immediately, ten microliters of sample suspension was placed on top, and a drop of the suspension was placed on a sheet of para-film. Second, a carbon-coated copper grid was floated on the drop for 10 s. Then, the grid was removed, and clean filter paper was used to drain the excess liquid. Third, ten microliters of 2% uranyl acetate, pH 7.0, was added to the grid for approximately 5 s. Fourth, the grid was drained using filter paper and allowed to dry for several minutes. Finally, the grid was examined using a JEM-1200 EX microscope (JEOL, Akishima, Japan) at 80 kilo electronvolts.

#### Scanning electron microscopy (SEM)

The scanned microscopy samples were prepared by preparing helium ionic slides with exosome samples. Accordingly, 10 µl of para-aldehyde was added to 10 µl of extracted exosomes and incubated for 20 min. Next, the samples were washed three times using PBS. Finally, exosome samples were dehydrated with 50%, 70%, and 100% ethanol and incubated for 2 h to dry for scanning microscopy.

### Statistical analysis

Two statistical comparisons were performed to compare the size distributions of the three types of extracellular vesicles. These comparisons employ one-way ANOVA and generalized linear models (GLMs) to determine if there is a significant difference between the datasets collected using the SEM and TEM techniques.

#### One-way ANOVA

One-way analysis of variance (ANOVA) is a statistical method used to compare the means of groups of datasets and determine if their means differ significantly. In this section, the test examines differences in the diameter means of the three extracellular vesicle (EXO–APV–NCB) cell types.

## Results and discussion

### Comparison between ultrafiltration and ultracentrifugation and the serial isolation methods purity

#### Western blot

**Fig. 1:**
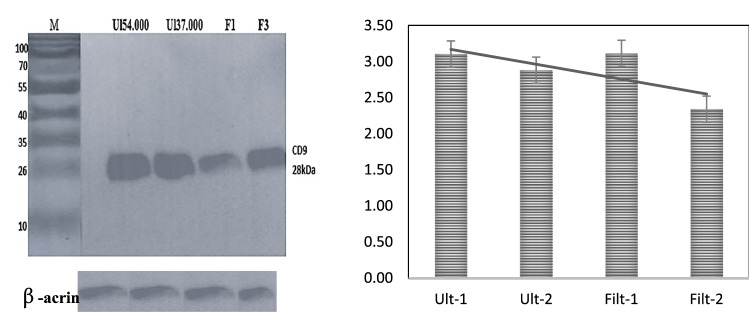
Band of 28 kDa of exosomes has been treated with CD9 of rabbit anti-mouse, column (i) Exosomes isolated using Ultracentrifugation, Ultrafiltration cryopreservation combination with higher yield (ii) Exosomes isolated using Ultracentrifugation alone (iii) Ultrafiltration devices alone (vi) the last band is β-actin control

On one hand, knowledge of EVs is, however, limited, mainly due to their sub-micrometer size and basic boundaries in methods applied for their characterization. On the other hand, exosome markers were massively characterized for improved isolation from the NCI 1975 lung cancer cell line. In this study, we investigated the purity and stability of exosome proteins after excessive isolation methods. Exosomes were isolated using ultracentrifugation followed by ultrafiltration, and then special preservation techniques improved exosome purity and yield concentration. This was detected using the western plot test. The products of ultracentrifugation techniques alone and ultrafiltration alone, compared with the combination of the three methods, were incredibly different in purity and concentration, as shown in Fig. 1(a) and (b).

**Fig. 2:**
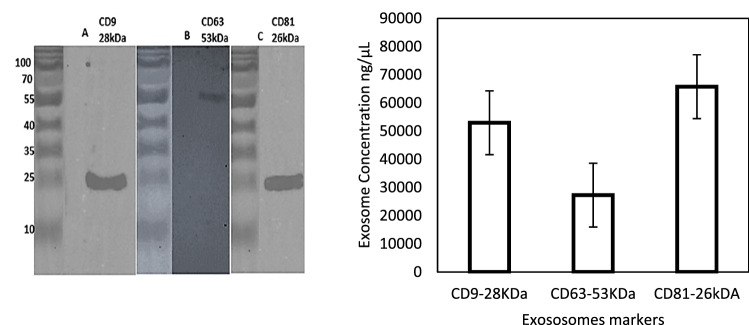
Exosomes protein lysate treated with 1:1000 **A** CD9 (26 kDa), **B** CD63 (55 kDa), and **C** CD81 (28 kDa) rabbit anti-human primary antibody, which reacted against secondary antibody rabbit anti-human primary antibody. Then, Goat anti-rabbit IGg secondary antibody. The band was then developed using Super Signal Chemiluminescent Substrates. **d** The histogram showed the different concentrations in each protein biomarker expressed on the NCI-1975 lung cancer cell line

Three conventional exosome markers (e.g., CD9, CD81, and CD63) were analyzed to characterize expression of the exosome's significant proteins (Omer 2018). A band of 26 kDa was observed when 10 µl (3 mg/ml) of exosome lysate was blotted against CD9 rabbit anti-human primary antibody, as shown in Fig. 1(i), (ii) and (iii). Likewise, a band of 28 kDa was observed when 10 µl of exosome lysate was blotted against CD81 rabbit anti-human primary antibody, as shown in Fig. 2. In contrast, as shown in the exact figure, a band of 55 kDa was observed when 10 µl of exosome lysate was blotted against rabbit anti-human. Expression (M. E. O 2014) of surface protein biomarkers is major prove of specific expression of NSCLC exosomes.

#### Morphology characterization using transmitted microscopy

**Fig. 3:**
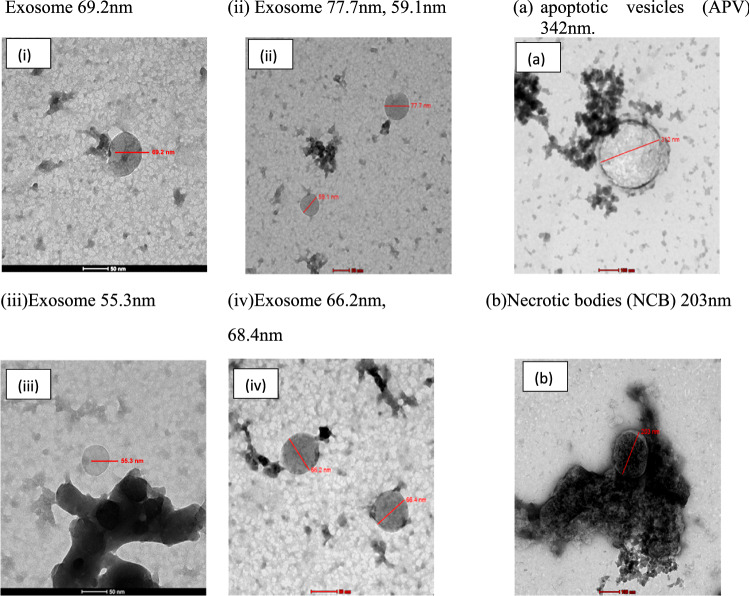
Transmitted Electron Microscope (TEM) was used for morphology characterization of exosome as shown (i, ii, iii, and iv) in diameter (30–100 nm). In compression with apoptotic vesicles (APV). Necrotic bodies (NCB) as shown in (**a**, **b**)

**Fig. 4:**
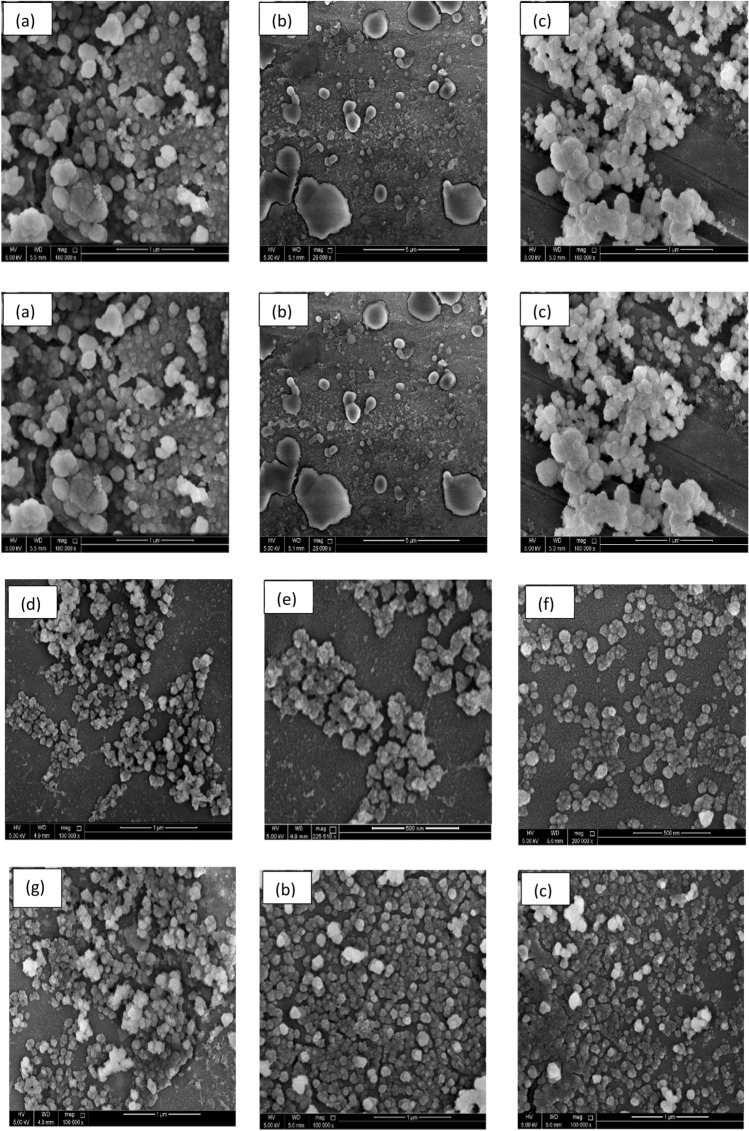
Characterization of circulated exosomes-derived NSCLC (NCI 1975) cells extracellular vehicles (EVs) and exosomes by scanning electron microscopic (SEM), which differentiated the shapes and sizes of exosomes and other types or extracellular vehicles. In Fig (**a**, **b**, **c**), apoptotic vesicles (AV). Necrotic bodies (NCB) in the range 300–500). In addition, **d**, **e** show exosomes were extracted using Ultrafiltration or alone, **f**, **c** or Ultracentrifugation alone, and **g**, **h**, **i** exosomes were extracted using Ultracentrifugation followed by Ultrafiltration or and kept in specific cryopreservation conditions. Variety of exosome sizes measured in the ranges between (30-100 nm)

Different sizes of micro- and nanoscale vesicles released by cells can be differentiated based on their size distribution and shapes. That differentiation is made easy using transmitted electron microscopy (TEM), which is considered a significant tool for characterizing the morphology of exosomes. However, scanning electron microscopy (SEM) is regarded as an advanced tool to further determine the shape and differentiate exosomes from the other nanovesicles induced in NCI 1975 conditioned media. The morphological characterization results showed that the exosomes were small vesicles. This finding has been observed by many researchers (Bonsergent et al. 2021; Arraud et al. 2014). Moreover, extracellular vesicles isolated from normal cells showed a round morphology consistent with exosome sizes and shapes isolated from normal cells. A similar observation was stated by Arraud et al. (2014); Doyle and Wang 2019), and (Holcar et al. 2020). In addition, the morphological shape of exosomes was computed to vary in size, ranging from 30 to 200 nm in diameter. A similar range was shown in Ref (Arraud et al. 2014; Jella et al. 2018). In contrast to exosomes, apoptotic vesicles and necrotic bodies isolated from NSCLC (NCI 1975) cells displayed irregular shapes and heterogeneous size distributions, as characterized by TEM and shown in Figs. 3 and 4. Additionally, TEM was used to describe the morphology of NSCLC (NCI 1975) cell exosomes, and a central depression was observed by TEM (Fig. 3).

### Statistical analysis

The extracellular vesicles and exosomes were compared using statistical analysis to reduce the errors of repeated ultracentrifugation steps that significantly impact the purity and solubility. However, exosome sizes and shapes usually differ because of excessive rounds of isolation methods. Therefore, statistical analysis that compared the sizes and shapes of exosomes demonstrated no significant differences in their sizes and shapes, as shown in Figs. 3 and 4. Additionally, as shown in Table 1, there was significant difference between the exosomes and other types of extracellular vesicles. Thus, the experiment was formulated as a test of the hypothesis in which H_0_ represents the null hypothesis proposing no significant difference in the mean diameters of extracellular vesicles, and H_1_ represents the opposite of H_0_ (e.g., the mean diameters of the extracellular vesicles are significantly different). Table 1 reports the settings and assumptions of the one-way ANOVA test.

**Table 1:** Experimental Setting of the one-way ANOVA test

Test Name:	One-way ANOVA
Software	Statistical Package for the Social Sciences (IBM®-SPSS®)
Purpose	Determine whether there are statistically significant differences between the means of the three cell types’ diameters
Item	Description
Factors	3 levels cell-type (EXO – APV – NCB)
Response	Means of the diameter
Null hypothesis	${\mathrm{H}}_{0}: {\mu }_{EXO}={\mu }_{APV}={\mu }_{NCB}$
Alter. hypothesis	${\mathrm{H}}_{1}$: Minimum of two diameter means are not equal
Model type	Fixed effect Unbalanced- one-way ANOVA
Assumptions	1. Equal variances across all Microscope types
	2. The diameter of each experiment is distinct from the diameter of any other experiment

ANOVA was conducted using IBM®-SPSS® software. This software is extensively utilized for advanced data management and analytics. The ANOVA results are shown in Table 2.

**Table 2:** One-way ANOVA test

Source of variation	DF	Adjusted SS	Adjusted MS	*F*-value	*P*-value
Cell-type	2	9,106,615	4,553,308	2210.89	0.000
Error	1076	2,216,009	2059		
Total	1078	11,322,625			

**Fig. 5:**
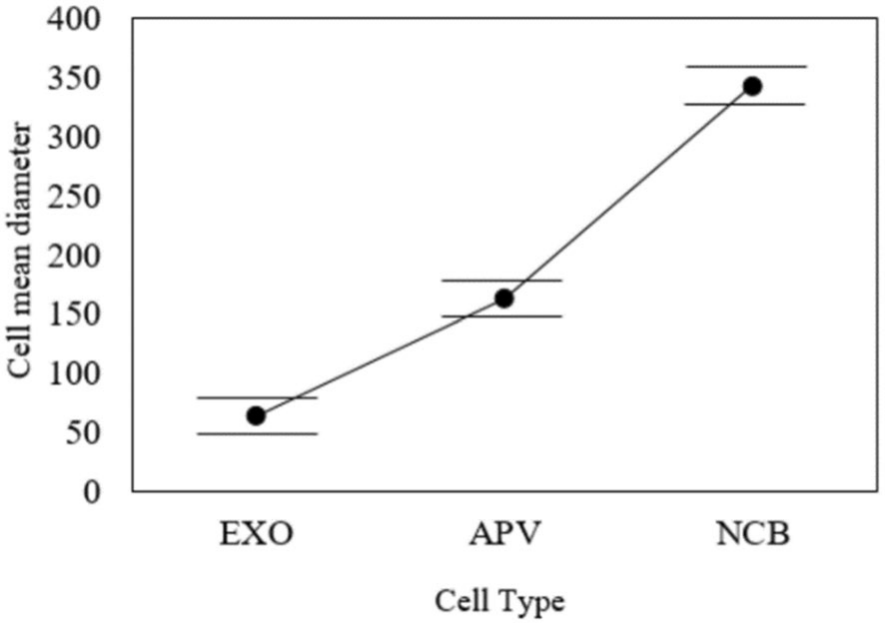
Interval plot of EXO, APV, and NCB

To determine whether there were any significant differences between the means of the cell types, we compared the *p* value to the significance level ($\alpha =0.05$). The *p* value quantifies the probability of obtaining the observed results, assuming that Hypothesis H_1_ is valid. To decide, we compare the *p* value with $\alpha$. If the *p* value $>\alpha$, then ${\mathrm{H}}_{0}$ should be accepted, and it is concluded that the mean diameters of the three cell types are assumed to be identical. If the *p* value $\le \alpha$, we should reject ct ${\mathrm{H}}_{0}$ and conclude that at least one cell type's diameter differs. However, the *p* value of 0.00 in Table 2 indicates that the means of the three cell types are significantly different. Table 3 and Fig. 5 report the three mean confidence interval comparisons. Figure 5 demonstrates no overlap between the mean 95% confidence intervals of the three cell types, indicating that the size distributions of extracellular vesicles are different. Similar results are described by Lee et al. (Lee et al. 2019).

**Table 3:** Factor statistics and intervals

Factor	*N*	Mean	StDev	95% CI
EXO	172	64.22	17.30	(57.43, 71.01)
APV	656	163.44	42.82	(159.96, 166.92)
NCB	251	343.61	62.09	(337.99, 349.24)

### GLM-based ANOVA Test

In Sect. GLM-based ANOVA Test, the one-way ANOVA disregards the effect of the microscopic method used to measure the diameter of the extracellular vesicle. Therefore, this section thus far employs the generalized linear model (GLM) to simultaneously examine the effect of cell types and microscopic techniques (SEM and TEM). The GLM is an ANOVA with multiple factors. Using the least squares method, the GLM characterizes the statistical relationship between a set of explanatory variables and a response variable (LSM). The settings and assumptions for the GLM-based.

IBM®-SPSS® software was utilized for GLM-based ANOVA. The factor information and GLM-based ANOVA results are presented in Tables 4, 5 and 6.

**Table 4:** Experimental setting of the GLM-ANOVA test

Item	Description
Factors	Factor 1: 3 levels cell-type (EXO–APV–NCB)
	Factor 2: Microscope Type (SEM–TEM)
Response	Means of the diameter
Null hypothesis	Cell-type:${H}_{0}: {\mu }_{EXO}={\mu }_{APV}={\mu }_{NCB}$
	Microscope: type ${H}_{0}:$ ${\mu }_{SEM}={\mu }_{TEM}$
Alter. hypothesis	Cell Ty ${\mathrm{H}}_{1}$: Minimum of two diameter means are not equal
	Microscope: type ${\mathrm{H}}_{1}$:${\mu }_{SEM}\ne {\mu }_{TEM}$
Model type	Fixed effect unbalanced- one-way ANOVA
Assumptions	3. Equal variances across all Microscope types
	4. Each experiment's diameter is distinct from any other experiment's diameter

**Table 5:** GLM-based ANOVA factor information

Factor	Type	Levels	Values
Cell-type	Fixed	3-levels	EXO-APV- NCB
Microscope-type	Fixed	2-levels	SEM-TEM

**Table 6:** GLM-based ANOVA test

Source	DF	Adjusted SS	Adjusted MS	*F*-value	*P*-value
Cell-type	2	9,102,400	4,551,200	2209.11	0.000
Microscope-type	1	1296	1296	0.63	0.428
Error	1075	2,214,713	2060		
Total	1078	11,322,625			

As the *p* value (0.000) for the cell-type test is less than the significance level (= 0.05), it can be concluded that there is a significant difference between the means of the diameter for the three cell-type levels. On the other hand, since the *p* value (0.428) for the microscope-type test is better than $\alpha$, there is no significant difference between the means of the microscope-type levels (SEM and TEM). The exosome membrane shape differs from the identical isolated samples depending on which EM techniques have captured the images. The TEM images show a characteristic central depression in the exosomes.

The SEM images show exosomes as round spheroids. These findings are also supported by refs Jella et al. (2018); Li et al. 2020), and (Mahgoub et al. 2019). The SEM images of exosomes, apoptotic vesicles, and necrotic bodies, shown in Fig. 3, show the morphologies, whole membrane structures, and differences between each nanovesicle type. These observations give an extra advantage to SEM as an effective alternative to TEM for direct imaging of extracellular vesicles based on improved sample processing methods. Similar statements were generated in Ref (Wu et al. 2015). Therefore, according to the GLM-based ANOVA test, there are significant differences between the cell types. Thus, Tukey's pairwise comparison was conducted to determine which means were different. Table 7 reports the results of the Tukey pairwise comparison using IBM®-SPSS® software. In our earlier methodology of the filtration device, as shown in Fig. 1(a, b and c), the exosome concentration was higher and purer in the one-step protocol. In Lobb (Gupta et al. 2018), these results also prove that a filtration device is more appropriate for collecting exosomes in better shape, with great purity and higher concentration (Yin et al. 2021). The use of filtration and ultracentrifugation gave a high yield of pure-quality exosomes and showed the same size as in Fig. 1.

**Table 7:** Grouping information using the Tukey method and 95% confidence

Cell-type	*N*	Mean	Grouping*
3	251	341.879	A
2	656	161.300	B
1	172	62.619	C
*Microscope-type*			
1	1008	190.857	A
2	71	186.342	A

*Means that do not share a letter are significantly different

Isolated exosomes are directly fixed on a metallic formvar grid and are observed after negative staining with uranyl acetate. Dispersing samples containing extracellular vesicles as a monolayer on the silicon substrate significantly improved the image quality. Furthermore, exosome diameters are shown in the TEM method NSCLC (NCI 1975) cell line. The size distributions of exosomes were not significantly different between the two EM methods (*p* value = 0.428 > 0.05). Based on the statistical analysis, one can conclude that there is no significant difference between the means of the microscope-type levels (SEM and TEM) (Wu et al. 2015).

Table 7 demonstrates that the three cell types have distinct group indices (no overlapping). The evidence supports the findings of the one-way ANOVA test. The average diameters of the three cell types differed significantly. In contrast, the microscope methods belong to the same group, "A." This finding indicates that these two methods, followed by cryopreservation under specific conditions, produce extracellular vesicles and exosomes with a constant diameter. The size distributions of exosomes purified from NSCLC (NCI 1975) cells at the indicated incubation times (12–48 h) with the parental NSCLC (NCI 1975) cells were assessed for significant differences by two-way and one-way ANOVA analysis.

These size distributions were similar regarding the incubation medium types and times. A change in exosome size distribution toward smaller sizes can be explained by the culture conditions increasing the cell's death, as observed by the extremity in the distribution of vesicles obtained after incubation in 37 °C in conditioning media or incubation at 37 °C for 48 h in serum-free media. Some advanced studies (Meng et al. 2020; Popowski et al. 2020) explained the changes in exosome cell sizes to the parental cell sources. This finding was also observed in the human HEK cell Line 293 T, which shrunk in size by 50% within eight days from 116 to 63 nm in diameter due to keeping the isolated exosomes in PBS at 4°C (Wu et al. 2015; Wu 2015). In different studies (Ghosh et al. 2014; Javeed et al. 2015), exosomes can be of different sizes due to long incubation hours. Accordingly, the two-step isolation followed by cryopreservation in specific conditions keeps the exosome protein surface (lipoprotein surface) free from crystallization.

## Conclusion

In this study, the NCI 1975 cell line was used as a source of exosomes. The isolation processes made it challenging to identify the optimal exosome isolation protocol. The exosome-isolated products were screened under TEM and SEM. As a result, the following conclusion can be drawn.

Although ultracentrifugation gives the exosome concentration yield, as shown in Fig. 1, the isolation product can be contaminated, as La Shu et al*.* (Shu et al. 2020) explained the difference in exosome purity after several rounds of ultracentrifugation even though the yield and concentration were high. Therefore, ultrafiltration using 300 kDa followed by 100 kDa resulted in the highest purity, and the least contaminated pellets appeared, reducing the possibility of contamination, as proven by La Shu et al*.* (Shu et al. 2021). Combining the two methods resulted in the collection of pure exosomes that were less contaminated and had a smaller yield than the usual amount yielded using ultracentrifugation alone, as shown in Fig. 1.

Furthermore, their cryopreservation is the last step of exosome isolation. The size of exosomes usually differs when stored at  – 20 °C or  – 80 °C for an extended period. It shrinks in size, and the lipoprotein walls start losing some of their content. This fact is demonstrated using morphological characterization that measures the exosome sizes and compares it with different types of extracellular vesicles by using two types of electron microscope, a scanning electron microscope (SEM) and a transmission electron microscope (TEM). These instruments were used to characterize the exosomes' original morphology and observe any change in the shapes and sizes.

In addition, it has been reported that considerable differences in producing and storing exosomes could change experimental observations of their purity and size. SEM imaging also proved the influence of cell culture media on exosome production, purity, and size storage conditions. However, in the statistical analysis of the cells, the size distributions of apoptotic vesicles (APVs) (450 nm) and necrotic bodies (NCBs) (280 nm) were substantially different from the size distributions of exosomes (*P* < 0.001). Moreover, the mean diameters of the three cell types examined under the two kinds of electron microscopy methods were analyzed using the GLM-NOVO test and showed no significant difference. The morphological analysis proved that the size distributions of extracellular vesicles in compression to exosome sizes were significantly different from exosomes, and the sizes were significantly constant.

Finally, up to 15-fold more exosomes were obtained using ultracentrifugation alone. While the purity concentrations of co-isolated using ultrafiltration soluble factors were adjusted for exosome yield, finding a greater than twofold increase in PD-L1-expressing exosomes is considered an outstanding achievement. Mechanistically, in the context of the immunomodulatory effects of exosomes, non-small cell lung cancer (NSCLC) cells secrete exosomes in the media with the help of endosomes in the cytoplasm (Mahgoub 2017; Chen 2021). Therefore, exosomes can be of different sizes due to prolonged incubation hours. Accordingly, the two-step isolation followed by cryopreservation in specific conditions keeps the exosome protein surface (lipoprotein surface) free from crystallization.

## Data Availability

The Data in this paper will be available when it will be requested.
